# Deep Learning: A Rapid and Efficient Route to Automatic Metasurface Design

**DOI:** 10.1002/advs.201900128

**Published:** 2019-04-19

**Authors:** Tianshuo Qiu, Xin Shi, Jiafu Wang, Yongfeng Li, Shaobo Qu, Qiang Cheng, Tiejun Cui, Sai Sui

**Affiliations:** ^1^ Department of Basic Sciences Air Force Engineering University Xi'an 710051 China; ^2^ School of Computer Science Xi'an Polytechnic University Xi'an 710048 China; ^3^ State Key Laboratory of Millimeter Waves Southeast University Nanjing 210096 China

**Keywords:** absorbers, autoencoders, deep learning, discrete cosine transform, metasurfaces

## Abstract

Metasurfaces provide unprecedented routes to manipulations on electromagnetic waves, which can realize many exotic functionalities. Despite the rapid development of metasurfaces in recent years, the design process of metasurface is still time‐consuming and computational resource‐consuming. Moreover, it is quite complicated for layman users to design metasurfaces as plenty of specialized knowledge is required. In this work, a metasurface design method named REACTIVE is proposed on the basis of deep learning, as deep learning method has shown its natural advantages and superiorities in mining undefined rules automatically in many fields. REACTIVE is capable of calculating metasurface structure directly through a given design target; meanwhile, it also shows the advantage in making the design process automatic, more efficient, less time‐consuming, and less computational resource‐consuming. Besides, it asks for less professional knowledge, so that engineers are required only to pay attention to the design target. Herein, a triple‐band absorber is designed using the REACTIVE method, where a deep learning model computes the metasurface structure automatically through inputting the desired absorption rate. The whole design process is achieved 200 times faster than the conventional one, which convincingly demonstrates the superiority of this design method. REACTIVE is an effective design tool for designers, especially for laymen users and engineers.

## 1 Introduction

Metasurfaces, composed of sub‐wavelength resonators in 2D plane,^1^, ^2^, ^3^ are capable of providing an unprecedented route to powerful control over electromagnetic (EM) waves in terms of propagation modes, polarization, and wave‐fonts.^4^, ^5^, ^6^, ^7^ Due to their unique EM properties, metasurfaces have attracted great attentions from engineers and researchers. Recently, many novel metasurfaces have been presented and many exotic functionalities can be realized, such as holography,^8^, ^9^ perfect absorption,^10^, ^11^ vortex beam generation,^12^, ^13^ flat lenses,^14^, ^15^ and some other functional interfaces.^16^, ^17^, ^18^, ^19^

However, conventional design process usually consists of model design, parameter sweeping, and optimization. The utilization of the optimization algorithm also requires tens or even hundreds of EM simulations, which is time‐consuming and computational resource‐consuming; moreover, designers have to spend a lot of time and pay much attention on modeling and optimizing. In addition, as EM field theory is the design basis, designers are required to be highly professional in this field, which prevents layman users from metasurface design according to actual demands. Besides, since the metasurface model is not adaptive, each realization of a new function asks for changes of the design model and the design ideas. Therefore, it is of great significance to figure out a rapid, efficient, and automatic metasurface design method, which is expected to be suitable for metasurface design with different functions.

Deep learning, a branch of machine learning, is an inter‐discipline which researches on how computers simulate and realize human learning patterns so as to acquire new knowledge or skills. Nowadays, deep learning methods have already made it come true that computers' inferential capability can be optimized by “studying” on previous datasets or experiences. Moreover, in terms of the known‐rule problems, there have been some outstanding inventions which verify that deep learning exceeds human in some ways. One of them is the astonished AlphaGo, an automatic Go robot, developed by Google in 2016.^20^, ^21^ By means of the deep learning method, convolutional neural network in precise, AlphaGo defeats many world‐famous Go players including the two tops Ke Jie and Lee Se‐dol, which results in the consensus in the Go field that AlphaGo has surpassed the top level of human Go ability. Other cases include the application in data mining where human are always prone to making mistakes, such as cancer prognosis and prediction,^22^, ^23^ automated text and image classification,^24^, ^25^ user behavior prediction,^26^, ^27^ etc. Whether machine learning or its branch deep learning, they both have already shown their achievements and efficiency in predicting and clustering. The basic idea of which is that, according to a set of given data, an algorithm is designed to find the rules between input data and output data; if the rules between input and output are not changed, once given another input, machine can predict its output automatically and rapidly. As deep learning has its natural advantages and superiorities in mining undefined rules automatically,^28^, ^29^ we therefore associated deep learning theory with our metasurface design, with the expectation that deep learning methods are capable of designing metasurfaces. This may result in a subversive breakthrough in the field of metasurface design, which enables the metasurface design to be rapid, efficient, and automatic. More importantly, there will be no professional theoretical requirements on designers so that engineers are only required to pay attention to their practical demands instead of to the complicate design process.

Inspired by this, in this paper, a rapid, efficient, and automatic metasurface design method named REACTIVE is proposed. In our method, we connect the metasurface structure with its EM properties through deep learning method, where we set up and train the deep learning model by a set of samples; the model is capable of finding out the inner rules between metasurface structure and its EM properties. The simulation result shows that our model can reach an average accuracy of 76.5% while for the top 30% samples, the accuracy rate will be higher than 90%. Afterward, the deep learning model is able to automatically generate the metasurface structure as the output, with any given design target of EM property as the input. As an example, we design a triple‐band metasurface absorber using the REACTIVE method. Once a design target of the metasurface is input into trained deep learning model, the structure of the metasurface will be generated automatically. The structure performs three absorption peaks at 6.1, 17.3, and 19.1 GHz with absorption rates of 67%, 93%, and 99.7%, respectively, which is in good accordance with the design target. The whole design process of REACTIVE is 200 times faster than the conventional one, and can reduce the computational quantities and improve accuracy of design results.

REACTIVE does not need the process of modeling, parameter sweeping, and optimizing, which simplifies the design steps under the premise of improving the efficiency and effectiveness. REACTIVE figures out the metasurface structure in a few seconds with no need for iteration in the EM simulation. Moreover, a well‐trained deep learning model is capable of adapting to a variety of design requirements. As long as the deep learning model is not changed, there will be no further training process required for new design targets. REACTIVE method makes the design process automatic, more efficient, less time‐consuming, and less computational resource‐consuming. According to the given design target, REACTIVE figures out the metasurface structure directly. It is an effective design tool for designers especially for layman users who have no professional knowledge on EM theory. Moreover, our method shows preferable portability which enables the realization of other metasurface functions such as frequency selective surface.^30^, ^31^ This paper is organized as follows: Section 2 describes the general design idea and the deep learning theories in details. Section 3 illustrates the simulation result from the aspect of data gathering, train model setting up, and an example of metasurface design. Section 4 comes to the conclusion where we summarize the REACTIVE method.

## 2 Theory and Design

### 2.1 Introduction of REACTIVE

Based on deep learning, a rapid, efficient, and automatic metasurface design method, named REACTIVE, is proposed in this paper. We associate the metasurface structure with its EM properties through deep learning method. In this part, we first introduce the structure of metasurfaces, followed by the overall REACTIVE design idea from the aspect of training and generating metasurface model.

#### 2.1.1 Structure of Metasurfaces

**Figure 1:**
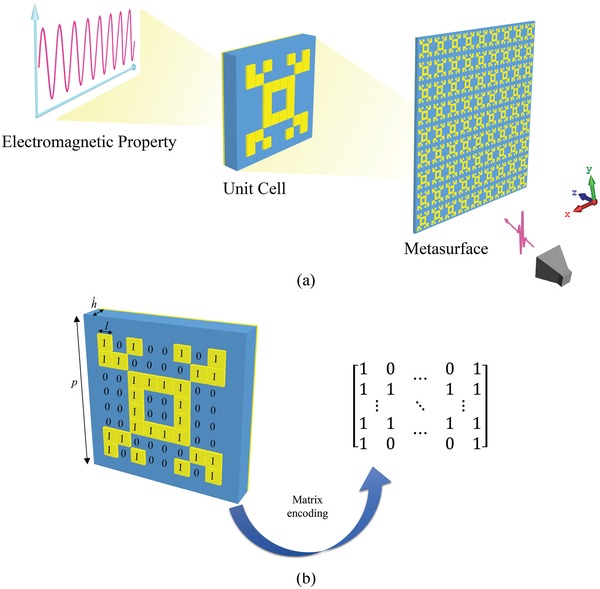
a) Schematic illustration of the metasurface structure; b) schematic illustration of the unit cell and matrix encoding method.

**Figure** **1** shows the geometry of the metasurfaces, consisting of three layers: a top cooper pattern layer, a dielectric layer, and a backing copper ground layer. The dielectric layer is an FR4 dielectric substrate with a thickness *h* = 1.5 mm, a dielectric constant ε_r_ = 4.2, and a loss tangent tanδ = 0.025. The thickness of the copper metallic patterns *t* is 0.035 mm. The top layer is composed of many unit cells, each of which can be divided into 8 × 8 lattices marked as “0” or “1.” The length of the lattices is *l* = 1.0 mm and the periodicity of unit cells is *p* = 10.0 mm. “1” lattice means that the area is covered by copper, whereas “0” lattice means that the area is blank. In this way, the structure of the unit cell can be encoded by a matrix.

It is apparent that there is a close relationship between the metasurface pattern matrices and the EM properties. Each pattern matrix corresponds to a set of *S*‐parameters. However, the number of matrix and coding methods is 2^64^, approximately 1.85 × 10^19^, the quantity of which is fairly huge. It will take thousand trillions of years to cover all the data, which is an impossible task under current calculation conditions. Besides, it is difficult to directly discover the rules between metasurface matrices and *S*‐parameters. Therefore, it is of great value to use deep learning design method to reduce the computational volume and to find out the optimal value. Moreover, deep learning is able to discover the rules between the lattices and EM properties, and to generate the metasurface structure automatically from *S*‐parameters. To describe the design principle more clearly, a twofold symmetrical structure, whose symmetry axes are along *x*‐axis and *y*‐axis on *x‐o‐y* plane, is introduced into the unit cell. Therefore, the unit cell can be described as a matrix of 4 × 4.

#### 2.1.2 Training Process of REACTIVE

In order to get a deep learning model, *S*‐parameters are regarded as input data while the metasurface pattern matrices are taken as the output data. The commercial EM simulation software CST Microwave Studio (MWS) is used to calculate the *S*‐parameters of the metasurfaces.

The training process of REACTIVE consists of data gathering, feature extraction, and metasurface pattern matrices matching. First, we pick up a series of matrices randomly and calculate their corresponding *S*‐parameters using CST MWS. The volume of data is determined through theoretical analysis and simulation which demonstrate the adequacy of 2000 sets of samples as training data. The pattern matrices are taken as output data and the simulated *S*‐parameters are taken as input data for the REACTIVE model; then, using the feature extraction method, the input data will be extracted as 64‐dimension features instead of the original 1000‐dimensional ones. Finally, it comes the matching part between extracted features and metasurface pattern matrices, where full connected multi‐layer perceptron (MLP) method, a kind of deep learning algorithm, makes it effective and realizable. The specific theories and their derivation will be illustrated in the following part.

#### 2.1.3 Design Process of the Metasurface

**Figure 2:**
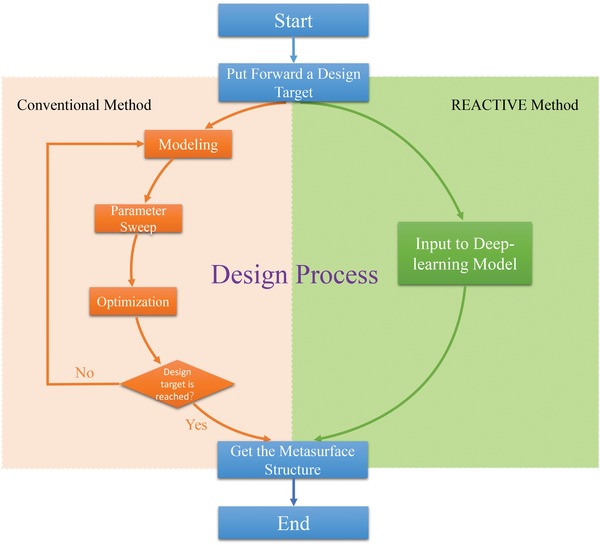
The contrastive flowchart of design process of REACTIVE method and conventional metasurface design method.

**Figure** **2** shows the flowchart of the design process of REACTIVE method. For the sake of comparison, the flowchart of conventional metasurface design method is also given. The design process of REACTIVE is illustrated as follows. After the deep learning model is trained successfully, we simply put forward a design target of *S*‐parameters and input them into the trained deep learning model, then the corresponding metasurface pattern matrix will be generated automatically as the output of trained deep learning model. In contrast, the design process of conventional method can be described as below. Once a design target of the metasurface is proposed, the designers should first build a metasurface structure according to EM field theory; then, parameter sweeping and optimizing are executed to obtain the “best available” values. If the “best available” values meet the requirements of design target, the design process ends; otherwise, a new structure will be built once more and the design procedure will restart until the requested results are obtained.

Comparing the REACTIVE method and conventional metasurface design method, we can clearly find that REACTIVE does not need the process of modeling, parameter sweeping, and optimization, which simplifies the design process and also improves the efficiency and effectiveness. Moreover, another superiority of REACTIVE is its ability of automatically generating the metasurface structure, so that engineers are able to concentrate their attention on their design targets, rather than the other detailed design process.

### 2.2 Theories and Methodology of REACTIVE

This part mainly illustrates the technical details of the training process of REACTIVE model. The training process can be divided into three parts: primary feature extraction, further feature extraction, and metasurface structure matching. For primary feature extraction, we apply cepstrum transformation to extract 200‐dimension features instead of the original 1000‐dimension ones; while in further feature extraction, Autoencoder‐based approach is used to extract features into 64 dimensions. In order to match extracted features with metasurface structure, we employ full connected MLP to get the connection.

#### 2.2.1 Primary Feature Extraction

Features are properties to describe or measure a certain object. Deep learning essentially deduces and concludes rules on the basis of feature sets. However, due to the rapid development of the big data technology, the dimension of features expands largely from tens to thousands, which leads to exponential increase of calculation quantities and difficulties, also known as the curse of dimensionality. Fortunately, according to relevant researches,^32^, ^33^ for a fixed learning task, a subset of given features, called relevant features, may be sufficient to describe the input data; while other features, named irrelevant features or redundant features, are dependent or just serve as pure noise which may introduce bias.^34^, ^35^ Hence, it is crucial to select or extract relevant feature subsets from features so as to speed up deep learning algorithms, improve predictor performance, and reduce the effect of curse of dimensionality. In order to reduce feature dimensionality, two main available approaches are feature selection and feature extraction; the former one aims at selecting relevant feature subsets from a given feature sets while the latter one transfers features into another feature space with a purpose of showing up less manageable features. On the whole, dimensionality reduction will result in effective improvement on the deep learning capacity, and it has been designed as an initial and crucial part of data preprocessing.

In the case of our task, the input data is the graphical representation of *S*‐parameters, the dimension for a single sample of which is more than a thousand. Observing from the input data graphic, it is similar to the acoustic signal to some extent with digital data representing *S*‐parameter scattering intensity at each discrete frequency step. As for acoustic signals, the extraction of the best feature representation is highly correlated with the better learning algorithm performance.^36^ Similarly, feature extraction is also necessary for our task. With relevant features extracted and irrelevant features removed, the dimension of features is reduced without loss of important information, so that a better performance can be achieved.

**Figure 3:**
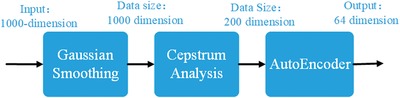
The flowchart of feature extraction in the REACTIVE method.

Due to the feature similarity between the acoustic signal and the input data in our task, we take the voice recognition methodology for reference. The overall process of our feature extraction method is shown in **Figure** **3**, after which every input data with 1000 dimension can be parameterized into a 64‐dimension feature vector.

Step1: Smoothing

Unstable voltage or electricity, EM wave caused by interference source may result in noise during the generation of input data. As these kinds of noises always follow Gaussian distribution, the utilization of Gaussian filter is an effective way of eliminating noises. Gaussian function is defined as

(1) $$f(x)\;\;=\;\;\frac{1}{\delta \sqrt{2\pi }}\exp^{-\frac{{\left(x-\mu \right)}^{2}}{2\delta^{2}}}$$

where *µ* represents the mean value while δ is the standard deviation. In our method, we set the length of Gaussian mask to be 5, the mean value 0, and standard deviation 1. In this case, the values of *f*(−2), *f* (−1), *f* (0), *f* (1), and *f* (2) compose the smoothing mask. After smoothing, the input data will be changed as

(2) $$x_{\mathrm{out}}(n)\;\;=\;\;\sum_{n=0}^{l-(\mathrm{mask}-1)}\sum_{i=0}^{\mathrm{mask}-1}\;\left(x(n\;\;+\;\;i)f\left(i\;\;-\;\;\frac{\mathrm{mask}\;\;-\;\;1}{2}\right)\right)$$

where *x*(*n*) and *f* (*i*) represents the input data and Gaussian mask, respectively; mask and *l* denote the length of Gaussian mask and input data, respectively.

Step2: Cepstrum transformation

The feature extraction process is applied after Gaussian smoothing. Considering that each sampled point of our input data carries its unique information, each single point should be regarded as an individual feature. Therefore, subset feature selection results in great loss of information. For voice recognition tasks, features are usually extracted from spectrogram by applying a series of transform, such as fast Fourier transform algorithm (FFT). As mentioned above, the input data in our task is of great similarity to acoustic signal, so that the methodology of acoustic feature extraction method Mel Frequency Cepstral Coefficients^37^ can be taken as reference. To be precise, we first reduce feature dimensionality by taking cepstum which is defined as the Fourier transform of the logarithm on frequency spectrum. The detail of our proposed cepstrum analysis is illustrated as follows.

For the input data, as the horizontal axis represents frequency, it can be regarded as the acoustic frequency spectrum defined in Equation (3)

(3) X(k)  =  H(k)E(k)

where *H*(*k*) is the spectrum envelop and *E*(*k*) is the spectrum detail. As what we want to extract is the information carried in *H*(*k*), we take a logarithm and play inverse FFT (IFFT) on *X*(*k*), so that the frequency spectrum will be changed into a pseudo‐frequency domain, where *H*(*k*) occupies the low pseudo‐frequency region and *E*(*k*) takes up the high pseudo‐frequency region.^38^ Hence, we only care about the low pseudo‐frequency and it represents the spectrum envelop. In practice, as the result of FFT is plural, to keep details as much as possible, we replace FFT function by discrete cosine transform (DCT), so that the result keeps in real domain. DCT transform is defined as below

(4) $$F(u)\;\;=\;\;c(u)\sum_{i=0}^{N-1}\;f(i)\cos\;\left[\left(\frac{(2i\;\;+\;\;1)\pi }{2N}u\right)\right]$$

(5) $$c(u)\;\;=\;\;\{\begin{array}{c} \sqrt{\frac{1}{N}},\;\;u\;\;=\;\;0\\ \sqrt{\frac{2}{N}},\;\;u\;\;\neq \;\;0 \end{array}$$

The details of the derivation process of DCT transform is shown in the Supporting Information.

**Figure 4:**
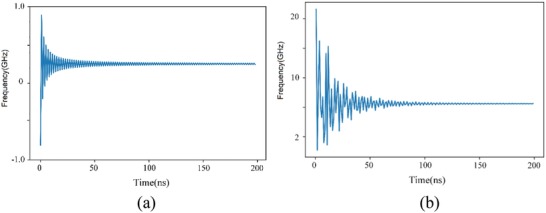
Graphics of DCT transform: a) AC coefficients of DCT transform, b) an example after applying DCT transform onto an input data.

Through analysis on the result of DCT transform, DC coefficient occupies the crucial part of information, AC coefficient suffers from attenuation, as shown in **Figure** **4**a where we plot the first 100 points of AC coefficient. Figure 4b presents an example after DCT transform. As expected, the core information concentrates in the low pseudo‐frequency region. The amplitude in the pseudo‐frequency domain attenuates with the increase of horizontal axis. As a matter of fact, the first 200 points represent more than 98% information in our task; hence, they are extracted as the initial extracted features.

#### 2.2.2 Autoencoder‐Based Further Feature Extraction

The next step of feature extraction comes to the deep learning method using the Autoencoder concept. It is a kind of neural network whose output data is the recovery of input by encoding and decoding in the hidden layer. The structure of Autoencoder consists of two parts, an encoder function *h = f*(*x*) and a decoder function *r = g*(*h*) that generates the reconstructed input data. However, if the Autoencoder simply deals with how to recover fixed inputs, namely, *g*(*f*(*x*)) *= x*, it will be of no use as it is nonexpandable to other inputs. Hence, it is necessary to impose constraints so that it only gets the approximation of inputs that enables acquisition of more effective properties, i.e., instead of the deterministic function, Autoencoder expands the concept of encoder and decoder to random mapping *P*_encoder(_ *_h_*_|_ *_x_*_)_ and *P*_decoder(_ *_x_*_|_ *_h_*_)._

**Figure 5:**
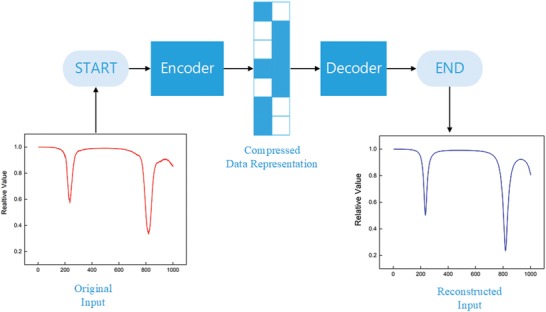
The flowchart of Autoencoder.

For our task, we set up an under‐complete Autoencoder, with lower dimension in the hidden layers than in the input layers, to further extract the features. The details are as follows. The flow chart of our proposed Autoencoder‐based feature extraction method is shown in **Figure** **5**. As our main purpose is to extract the more relevant features, we take down the decoder part of the Autoencoder model and reserve the output data of the encoder part. From the concept of under‐complete Autoencoder, the dimension of output from the hidden layer is lower than that of the input layer. Therefore, the learning process will force the encoder part to capture the most salient features of the input data, which results in a further reduction of feature dimensionality.

The main component of our Autoencoder structure is named as artificial neural network or MLP.^39^ The illustration of the information transmission through MLP layers is shown in Section S2 in the Supporting Information. Therefore, the feedforward transmission process into any layer of an MLP model can be expanded as follows

(6) $$\left\{ \begin{array}{l} y_{m}^{\left(n\right)}\;\;=\;\;f(u_{m}^{\left(n\right)})\\ u_{m}^{\left(n\right)}\;\;=\;\;\sum_{i\in L_{m-1}}w_{m}^{\left(ni\right)}y_{m-1}^{i}\;\;+\;\;b_{m}^{\left(n\right)}\\ y_{m}\;\;=\;\;f(u_{m})\;\;=\;\;f(W_{m}y_{m-1}\;\;+\;\;b_{m}) \end{array}\right.$$

where $w_{m}^{ni}$ and $b_{m}^{n}$ denote the coefficient and bias of the *n*th node on the *m*th layer, respectively; *f* (*) is named as the activation function, which implements the nonlinear mapping from input to output.

After constructing the model, the output data is generated on the basis of the parameters $w_{m}^{ni}$ and the input data. Then, it comes to the parameter updating problem, as multi‐layer structure is uncapable of updating parameters by means of the middle layers. As a matter of fact, the input and output of the middle layers are not accessible, so that the middle layers are always called hidden layers. Loss functions are defined to measure the difference between predicted value and true output value. Here, we use back‐forward propagation of the loss to estimate parameters. To be precise, for the last layer in the network, namely, the output layer, the loss function can be defined as

(7) $$L(w_{11},w_{12},\ldots ,w_{ij},\ldots w_{mn})\;\;=\;\;\frac{1}{2}{\left(o_{i}\;\;-\;\;y_{i}\;(w_{11},w_{12},\ldots ,w_{ij},\ldots w_{mn})\right)}^{2}$$

where **o**_*i*_ represents the expected output vector and **y** *_i_*(*w*_11_, *w*_12_,…, *w_mn_*) represents the predicted output vector.

For a given training set, the input vector *x_i_* and the expected output vector **o**_*i*_ can be regarded as constant. Hence, the loss function is only dependent on the weight coefficients *w_mn_*. The target of our task is to make the loss function as small as possible, we deduce the *w_mn_* updating process in Section S3 of the Supporting Information and we get the updating formula of

(8) $$w_{ij}\;\;\leftarrow \;\;w_{ij}\;\;+\;\;\Delta w_{ij}\;\;=\;\;w_{ij}\;\;-\;\;\eta \;\;\frac{\partial L(w_{ij})}{\partial w_{ij}}$$

where η is the updating rate defined by ourselves. The larger the η is, the faster the *w_ij_* changes.

It is not the good performance on training set but that on testing set matters, so the generalization ability is a crucial part of learning model design. For instance, overfitting happens when deep learning model fits the training data very well but fits the testing data badly. Data noise, insufficiency of training data, and model complexity are the main causes of the overfitting phenomenon. The former two can be effectively controlled during the data gathering process while regularization, a modification on loss function to enable the reduction of generalization error but not training error, is available to deal with model complexity. Many researches have been done on the regularization methods.^40^, ^41^, ^42^ In our method, L2 norm penalty, a kind of parameter norm penalty methods, is introduced. The loss function is changed and is defined as

(9) $$L(w_{11},w_{12},\ldots ,w_{ij},\ldots w_{mn})\;\;=\;\;\frac{1}{2}\;\;{\left(o_{i}\;\;-\;\;y_{i}(w_{11},w_{12},\ldots ,w_{ij},\ldots w_{mn})\right)}^{2}\;+\;\lambda \sum_{i\in L_{m-1},j\in L_{m}}w_{ij}^{2}$$

The derivation process of the parameter estimation and update are described in Section S4 of the Supporting Information and the updated formula of parameter *w_ij_* is changed as

(10) $$w_{ij}\;\;\leftarrow \;\;w_{ij}\;\;+\;\;\Delta w_{ij}\;\;=\;\;w_{ij}\;\;-\;\;\eta \frac{\partial L(w_{ij})}{\partial w_{ij}}\;\;=\;\;w_{ij}\;\;-\;\;\eta \;(-(o_{m}\;\;-\;\;y_{m})\;f\prime \;(u_{m})\;y_{m-1}^{i}\;\;+\;\;2\lambda w_{ij})$$

#### 2.2.3 Metasurface Structure Matching

After two steps of feature dimensionality reduction, the features for each sample have become 64‐dimensional ones, rather than the initial 1000 dimension. The matching problem is also converted from 1000‐dimension features and metasurface structure into 64‐dimension features and metasurface structure. From the aspect of deep learning method, the difficulty and accuracy of matching has been improved effectively, as the last layer of deep learning model, which controls the matching between features and targets, is fully connected.

The structure and theory of the matching part keeps the same as MLP while the activation function is changed. In neural network, each layer is comprised of many neuron nodes that receive information as input from the former layer and generate output for the next layer. Signal transmission process among neuron nodes is extracted as the mathematical model. The output from upper nodes and the input from lower nodes require a function mapping named as activation function. The activation function is capable of generating a nonlinear relationship between the input and output. To be precise, with the help of the activation function, the relationship between input and output can be written as

(11) $$y_{\mathrm{out}}\;\;=\;\;g(x_{\mathrm{in}})$$

where *g*(*·*) is a nonlinear function. From the explanation above, it is clear to verify that the activation function makes it possible to deal with nonlinear structure. Then, we must solve the problem of selecting appropriate activation function. As for our task, the target is zeros and ones from the aspect of digital signals, and the final outputs are limited to the range of (0,1). Therefore, we choose the sigmoid function as our activation function, the definition of which is

(12) $$S(x)\;\;=\;\;\frac{1}{1\;\;+\;\;e^{-x}}$$

Sigmoid function is capable of dealing with any output in (0,1). For the large negative inputs, it outputs 0; while for the S′ (x)  =  S(x)  (1  −  S(x)) large positive inputs, the output is 1. Besides, the derivative of sigmoid is easy to be calculated, so that it simplifies the weight coefficient updating process. Using the sigmoid function, the final output can be positively restricted between 0 and 1, in accordance with our targets.

Moreover, to deal with the overfitting problem, we not only use L2 regularization to change loss function, but also introduce dropout layer into REACTIVE model. It is a trick to avoid overfitting during training proposed by Hinton,^43^ it stops the coefficients in the hidden layer randomly so that it prevents the dependency of coefficients updating on conjunct effect of fixed hidden nodes. Hinton demonstrated many experiments to show the effectiveness of Dropout layer.

## 3 Simulation

## 3.1 Data Gathering

In order to train the deep learning model, a large number of metasurface pattern matrices and the corresponding *S*‐parameters are required to establish the dataset. Here, we use the metasurface model shown in Figure 1. *y*‐polarized wave illuminates the surface along the +*z* axis. The pattern matrix determines the structure of the metasurface, and the latter determines the *S*‐parameters and EM properties. Two thousand sets of random matrices are generated using “rand” function in MATLAB. And then metasurface structures represented by these matrices are input into the CST MWS in order to calculate the *S*‐parameters of the metasurface. Simulations were performed with unit cell boundary condition in *x* and *y* direction and open boundary condition in the *z* direction.

We set up the dataset, in which *S*_11_ is the input of the training model and the matrix of 4 × 4 is the output of the training model. Generally, we generate 2100 pairs of *S*‐parameter and metasurface pattern matrices to form our datasets; in more detail, we take 95% of the dataset as training set and the rest 5% as testing set. Therefore, there are 2000 training samples and 100 testing samples. Normally, in terms of the volume of training data in deep learning, we care more about the ratio between number of samples and number of features for each sample rather than the number of samples only. If we want to extract two features from 50 samples, it is namely to fit a quadratic function through 50 samples during training process. It is obvious that the number of samples is sufficient and the ratio between samples and features is 25. As for our task, we would like to fit 64‐dimension features though 2000 sets of samples, the ratio between number of samples and number of features is 31.25 which should be enough from the aspect of theoretical analysis.

### 3.2 REACTIVE Model Building and Training

The REACTIVE model is built under Window10 operation system. The configuration of computer is Intel(R) Core(TM) i5‐8250U CPU @ 1.6GHz to 1.8 GHz/8GB/256G SSD. Deep learning algorithm is realized on the Anaconda platform with python version 3.6.1. Tensorflow and Keras framework are also used to set up the model.

#### 3.2.1 Parameter Setting

**Figure 6:**
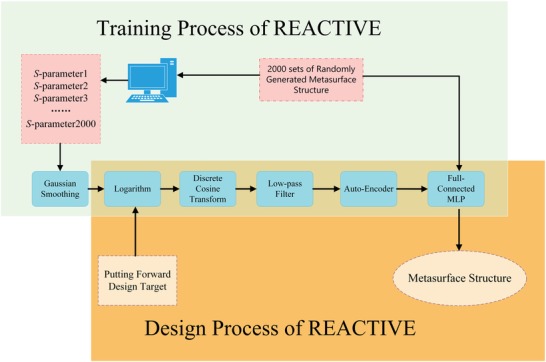
The flowchart of REACTIVE method.

**Figure 7:**
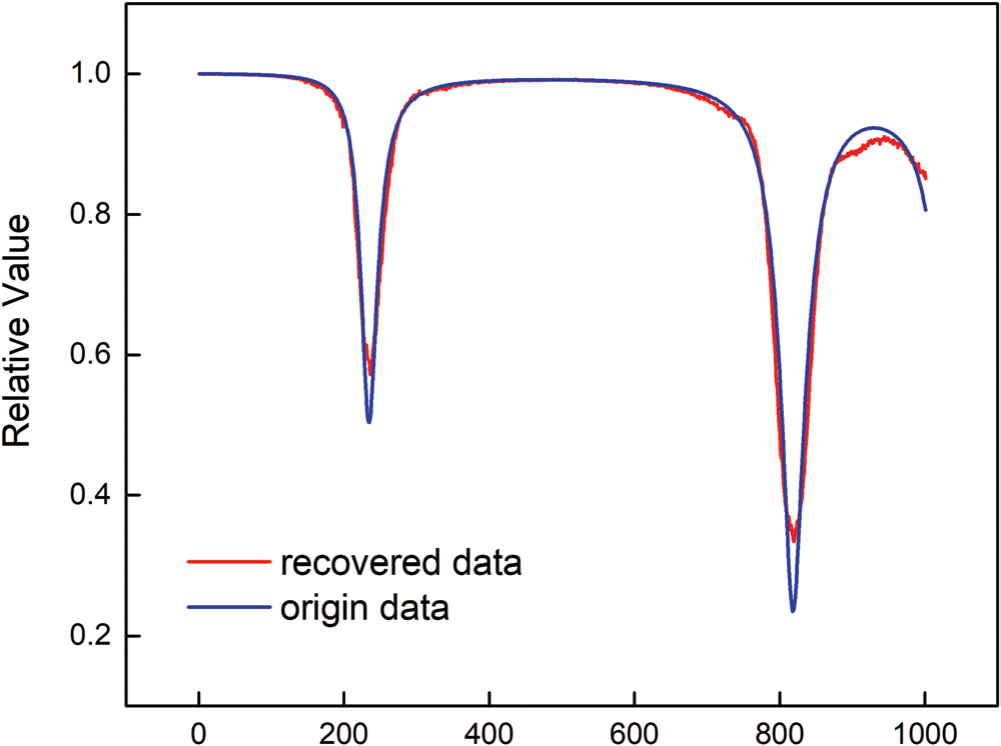
The comparison between original input data and the recovered data after applying Autoencoder.

The flowchart of REACTIVE method is shown in **Figure** **6**. We first apply DCT transform onto the datasets, and then apply the logarithm and low‐pass filter operation. After this, we only extract the first 200 points as representation and they can recover to original input with an accuracy of 98%. Second, we further extract features on the basis of under‐complete Autoencoder, where we set the batch size as 64, regularization method as L2 norm, and the activation function as sigmoid function. Moreover, for feature extraction, the loss function is defined as

(13) $$\mathrm{MAE}\;\;=\;\;\frac{1}{N}\;\;\sum_{i=1}^{N}\;\left|\left(f_{i}\;\;-\;\;y_{i}\right)\right|$$

where *f_i_* and *y_i_* represent the predicted value and the true value, respectively. **Figure** **7** shows the result before and after using Autoencoder. By comparing its Euclidean distance, it can be found that it keeps about 97% similarity to the original input with 64‐dimension data, rather than the initial 1000 dimension. According to the simulation result, our proposed feature extraction method is capable of extracting features effectively and efficiently with the feature size decreased to 6.4%.

After this, the features have been reduced from the initial 1000 dimension down to 64 dimensions. Then, it comes to the structure matching process using the fully connected MLP, where we choose 95% of the dataset as the training set and other 5% as the test set. In this part, all parameters are not changed but the loss function. It is defined as

(14) $$\mathrm{MSE}\;\;=\;\;\frac{1}{N}\;\;\sum_{i=1}^{N}\;{\left(f_{i}\;\;-\;\;y_{i}\right)}^{2}$$

where *f_i_* and *y_i_* share the same meaning as defined in MAE (in Equation (13)).

#### 3.2.2 Test and Comparison

**Figure 8:**
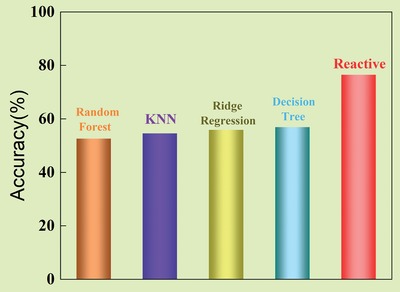
The comparison between REACTIVE and some other classical machine learning algorithms in terms of accuracy.

**Figure** **8** shows the result of accuracy rate of the REACTIVE method. For the sake of comparison, we also test on some traditional and classical machine learning methods, such as Decision Tree,^44^ Random Forest,^45^ Ridge Regression,^46^ and KNN (K‐nearest neighbors).^47^ It is clear to see that REACTIVE outperforms these classical machine learning methods, with an average accuracy of 76.5%. Especially, for the top 30% samples, the accuracy rate can reach as high as 90%. To be more specific, with the accuracy rate of 90%, our method is capable of correctly predicting 15 of 16 metasurface structural lattices.

In practice, as deep learning model only makes prediction about probabilities, it may not be precise enough to reach 100% accuracy, so further optimization process may be needed. Even so, the value calculated by deep learning is closer to the optimal value, which can also reduce the amount of calculation and speed up the design process.

### 3.3 Metasurface Design

**Figure 9:**
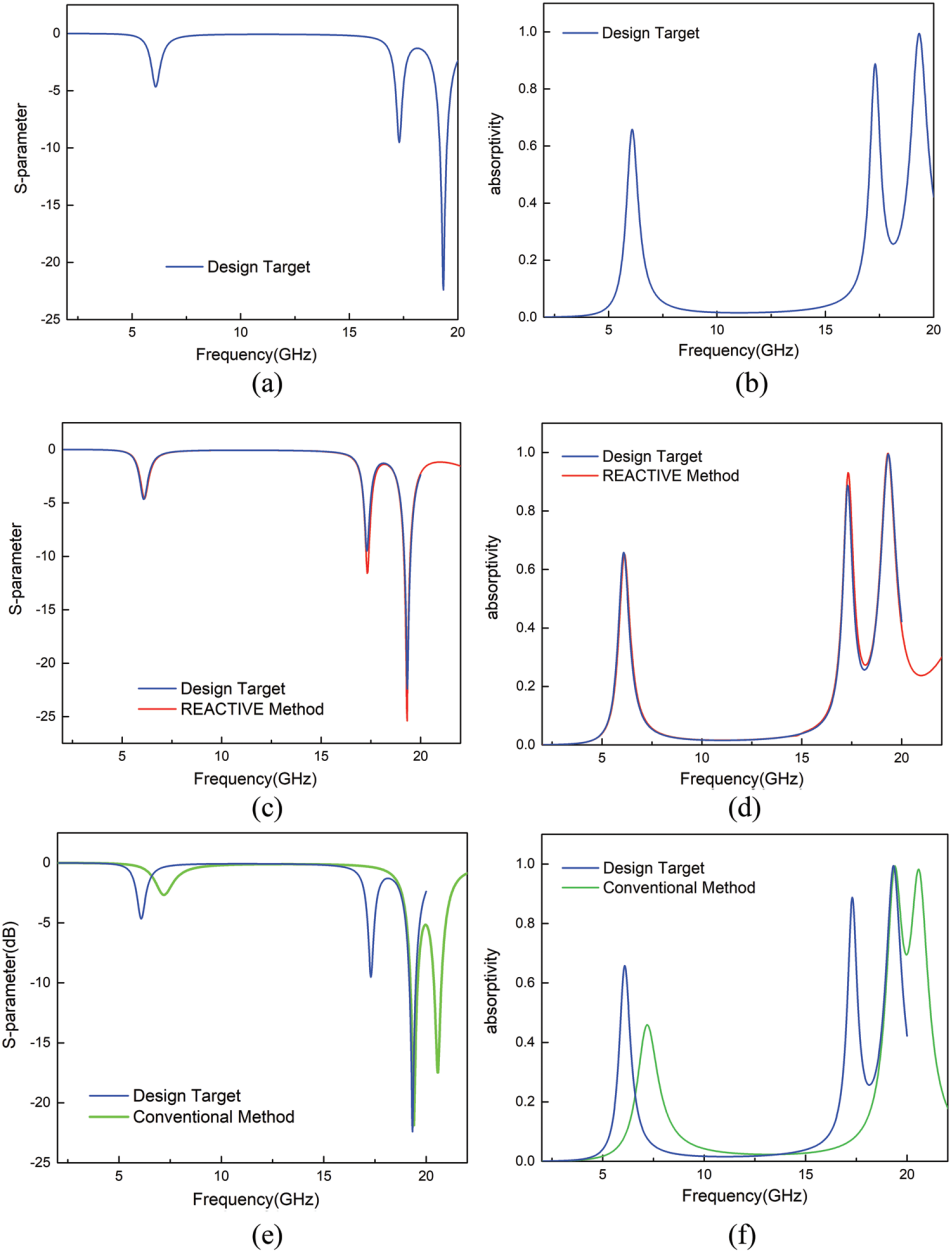
The comparison of Reflection coefficient *S*_11_ a) design target c) REACTIVE method e) conventional method and the comparison of absorption rate b) design target d) REACTIVE method f) conventional method.

As an example, we design a triple‐band metasurface absorber using the REACTIVE method. The desired *S*‐parameter and absorption rate are shown in **Figure** **9**a,b. Because of the absence of transmission, the absorption formula can be calculated by

(15) $$A\;\;=\;\;1\;\;-\;\;{\left|S_{11}\right|}^{2}$$

where *S*_11_ is the reflection coefficient. The absorptivities are 65%, 90%, and 99% at 6.1, 17.3, and 19.3 GHz. Meanwhile, the metasurface realizes total reflection characteristics in other bands.

In order to obtain the expected EM properties, we input the desired *S*‐parameter into the trained deep learning model and the matrix of metasurface structure is generated automatically through the *S*‐parameter. The optimal matrix of metasurface structure obtained according to REACTIVE method is

$$\left[\begin{array}{cccc} 1 & 0 & 1 & 0\\ 1 & 1 & 0 & 0\\ 0 & 0 & 1 & 1\\ 0 & 0 & 1 & 0 \end{array}\right]$$

The structure represented by this matrix is re‐entered into the CST MWS to calculate reflection coefficient *S*_11_ and absorption rate. From the curves shown in Figure 9c,d, it can be concluded that the structure performs three absorption peaks at 6.1, 17.3, and 19.3 GHz with absorption rates of 67%, 93%, and 99.7%, respectively, in good accordance with the design target.

For comparison, we design an absorber with the same function using conventional design method.^48^ In order to facilitate the comparison with REACTIVE method, we adopt the same structure as shown in Figure 1. We choose all “1” lattices as the initial value which means a 6 mm × 6 mm cooper patch is on the top layer. To achieve the design targets, *S*‐parameters in Figure 9a are set in the optimizer of CST MWS as the optimization goals.

The optimized absorption rate and *S*_11_ results are shown in Figure 9e,f. The absorptivities are 46%, 99.3%, and 98.2% at 7.2, 19.4, and 20.6 GHz. The results have frequency deviations compared with the design target. It is obvious that the result of the REACTIVE method is closer to the expected value than that of the conventional method.

**Figure 10:**
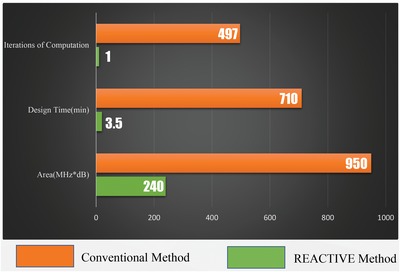
The comparison in terms of design time, iterations of computation, and area between computed *S*‐parameter and target *S*‐parameter.

To illustrate the advantages of REACTIVE more intuitively, as shown in **Figure** **10**, we compare the conventional method and REACTIVE in terms of design time, iterations of computation in CST, and area between computed *S*‐parameter and target *S*‐parameter. The design time of the REACTIVE method and conventional method is 3.5 min and 710 min, respectively. Meanwhile, the iterations of computation are one time and 497 times, respectively. The REACTIVE design process achieves nearly 200 times faster than the conventional one, which certifies that the REACTIVE method is more efficient and effective; additionally, it reduces the design difficulty.

We further compute the area between computed *S*‐parameter and target *S*‐parameter from 2 to 20 GHz, which reflects the accuracy of the design results. It is obvious that the result accuracy of REACTIVE method is much higher than that of conventional method.

Therefore, it can be concluded that the REACTIVE method is better than the conventional design method, either from the aspect of computational iterations, design time consumption, or result accuracy. Hence, the results validate the REACTIVE design method. The REACTIVE method provides an efficient way for 2D metasurface design in a variety of application environments.

## 4 Conclusion

In this paper, we propose a new metasurface design method named REACTIVE. Deep learning methodology is introduced into our method, which enables the automatic matching between metasurface structures with EM properties. Once a design target of the metasurface is input into trained deep learning model, the structure of the metasurface will be generated automatically. The REACTIVE method is able to reach as high as 90% accuracy in top 30% samples concerning the metasurface structure prediction. As an example, we use REACTIVE method to generate a tri‐band absorber. The consistency of the absorption characteristic and design target demonstrates the effectiveness of REACTIVE method. In the design process of the absorber, the REACTIVE method is better than the conventional design method from the aspects of computational iterations, design time consumption, and results' accuracy.

Compared with the conventional method, the automatic REACTIVE method shows a significant improvement on efficiency, an acceleration of design process as well as an obvious reduction on both computational and man‐powered resources. Besides, the trained model is suitable for new functions such as frequency selective surface without additional trainings. Moreover, this method has no requirements on professional knowledge in the area of EM theory and metamaterials. REACTIVE provides designers, especially the layman users and engineers, an effective design tool using which they are only required to concentrate on their design targets.

## Conflict of Interest

The authors declare no conflict of interest.

## Supporting information

Supplementary
