# Supplementary material for: Deep Learning: A Rapid and Efficient Route to Automatic Metasurface Design
Source: Adv Sci (Weinh). 2019 Apr 19;6(12):1900128. doi: 10.1002/advs.201900128 (PMC6662056; doi:10.1002/advs.201900128)
Supplement: Supplementary file 1 — Supplementary [file ADVS-6-1900128-s001.pdf]

Copyright WILEY-VCH Verlag GmbH & Co. KGaA, 69469 Weinheim, Germany, 2019.

Supporting Information

for *Adv. Sci.,* DOI: 10.1002/advs.201900128

Deep Learning: A Rapid and Efficient Route to Automatic Metasurface Design

Tianshuo Qiu, Xin Shi, Jiafu Wang, Yongfeng Li, Shaobo Qu, Qiang Cheng, Tiejun Cui, and Sai Sui*

Supporting Information

Deep learning: a rapid and efficient route to automatic metasurface design

Tianshuo Qiu, Xin Shi, Jiafu Wang, Yongfeng Li, Shaobo Qu, Qiang Cheng, Tiejun Cui and Sai Sui*

S1: Derivation of DCT transformation

We deduce the DCT transformation as follows. Firstly, the input data can be represented as

$$X(k) = H(k)E(k) \tag{1}$$

Secondly, we apply logarithm onto the input data, and get

$$\log X(k) = \log H(k) + \log E(k) \tag{2}$$

Finally, by applying DCT transform, we get

$$\begin{aligned} \mathrm{DCT}[X(k)] &= c(u)\sum_{k=0}^{N-1}[\log H(k) + \log E(k)]\cos[\frac{(2k+1)\pi}{2N}u] \\ &= c(u)\sum_{k=0}^{N-1}\log H(k)\cos[\frac{(2k+1)\pi}{2N}u] + c(u)\sum_{k=0}^{N-1}\log E(k)\cos[\frac{(2k+1)\pi}{2N}u] \end{aligned} \tag{3}$$

As mentioned in the manuscript, we care for the low pseudo-frequency that represents the spectrum envelop. *E(k)* represents the detail that changes near 0, so we omit the influence of *E(k)* in this step. Then, we get

$$\begin{aligned} \mathrm{DCT}[X(k)] &= c(u)\sum_{k=0}^{N-1}\log H(k)\cos[\frac{(2k+1)\pi}{2N}u] \\ &= \sqrt{\frac{1}{N}}\sum_{k=0}^{N-1}\log H(k) + \sqrt{\frac{2}{N}}\sum_{k=0}^{N-1}\log H(k)[\sum_{u=0}^{N-1}\cos\frac{(2k+1)\pi u}{2N}] \end{aligned} \tag{4}$$

The first term in the right of Equation 4 has nothing to do with parameter u, and it is called DC coefficient while the second term is called AC coefficient.

For AC coefficient, according the Euler formula

$$e^{j\theta} = \cos\theta + j\sin\theta \tag{5}$$

we further deduce the formula below

$$\begin{aligned}
\mathrm{DCT}[X(k)] &= \sqrt{\frac{1}{N}}\sum_{k=0}^{N-1}\log H(k) + \sqrt{\frac{2}{N}}\sum_{k=0}^{N-1}\log H(k)[\sum_{u=0}^{N-1}\cos\frac{(2k+1)\pi u}{2N}] \\
&= \sqrt{\frac{1}{N}}\sum_{k=0}^{N-1}\log H(k) + \sqrt{\frac{2}{N}}\sum_{k=0}^{N-1}\log H(k)[\sum_{u=0}^{N-1}\mathrm{Re}(e^{j\frac{(2k+1)\pi u}{2N}})] \\
&\approx \sqrt{\frac{1}{N}}\sum_{k=0}^{N-1}\log H(k) + \sqrt{\frac{2}{N}}\sum_{k=0}^{N-1}\log H(k)\,\mathrm{Re}(\frac{e^{\frac{\pi j(2k+1)}{2}}-1}{e^{\frac{\pi j(2k+1)}{2N}}-1})
\end{aligned} \tag{6}$$

S2: Illustration of multi-layer perceptron(MLP) structure

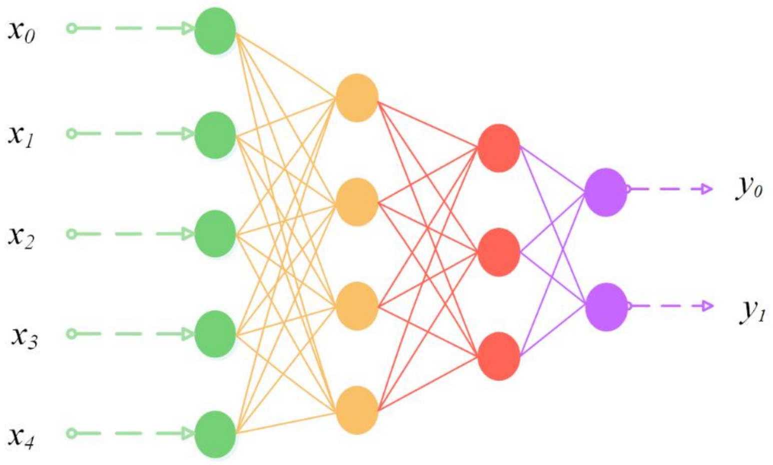

Figure S1. The structure of typical multi-layer perceptron (MLP) model

Figure S1 shows a typical MLP model, where the information is transmitted from the first layer to the last one. Take Figure S1 as an example, there are four layers in this model with five inputs and two outputs; besides, each layer has its input and output that are closely connected with former and latter layer by coefficients w_j^i .For

layer 2, the output should be

$$\begin{cases} y_2^{(1)} = f(\sum_{i=1}^{n} w_2^{1i} x_i) = f(w_2^{11}x_1 + w_2^{12}x_2 + w_2^{13}x_3 + w_2^{14}x_4 + w_2^{15}x_5) \\ y_2^{(2)} = f(\sum_{i=1}^{n} w_2^{2i} x_i) = f(w_2^{21}x_1 + w_2^{22}x_2 + w_2^{23}x_3 + w_2^{24}x_4 + w_2^{25}x_5) \\ y_2^{(3)} = f(\sum_{i=1}^{n} w_2^{3i} x_i) = f(w_2^{31}x_1 + w_2^{32}x_2 + w_2^{33}x_3 + w_2^{34}x_4 + w_2^{35}x_5) \\ y_2^{(4)} = f(\sum_{i=1}^{n} w_2^{4i} x_i) = f(w_2^{41}x_1 + w_2^{42}x_2 + w_2^{43}x_3 + w_2^{44}x_4 + w_2^{45}x_5) \end{cases} \tag{7}$$

The corresponding matrix expression is

$$\mathrm{y}_2 = \begin{bmatrix} y_2^{(1)} \\ y_2^{(2)} \\ y_2^{(3)} \\ y_2^{(4)} \end{bmatrix} = f\left(\begin{bmatrix} w_2^{11} & w_2^{12} & w_2^{13} & w_2^{14} & w_2^{15} \\ w_2^{21} & w_2^{22} & w_2^{23} & w_2^{24} & w_2^{25} \\ w_2^{31} & w_2^{32} & w_2^{33} & w_2^{34} & w_2^{35} \\ w_2^{41} & w_2^{42} & w_2^{43} & w_2^{44} & w_2^{45} \end{bmatrix}\right)\begin{bmatrix} x_1 \\ x_2 \\ x_3 \\ x_4 \\ x_5 \end{bmatrix} \tag{8}$$

For layer 4, the matrix expression of the output should be

$$\mathrm{y}_{out} = y_4 = f\left(\begin{bmatrix} w_4^{11} & w_4^{12} & w_4^{13} \\ w_4^{21} & w_4^{22} & w_4^{23} \end{bmatrix}\right)\begin{bmatrix} y_3^{(1)} \\ y_3^{(2)} \\ y_3^{(3)} \end{bmatrix} \tag{9}$$

S3: Derivation of coefficients updating in MLP

The target of MLP is to make to loss function as small as possible. For the loss function which is defined as

$$L(w_{11}, w_{12}, ..., w_{ij}, ...w_{mn}) = \frac{1}{2}(\mathbf{o}_i - \mathbf{y}_i(w_{11}, w_{12}, ..., w_{ij}, ...w_{mn}))^2 \tag{10}$$

$\mathbf{o_i}$ represents the expected output vector and $\mathbf{y_i(w_{11}, w_{12}, ..., w_{mn})}$ represents the predicted output vector, they can be regarded as constant.

In order to get the minimum value of Equation 10, we calculate the partial derivatives of $L(w_{11}, w_{12}, ..., w_{ij}, ...w_{mn})$ with respect to w_{ij}. According to Equation 10

and the chain rule, for any layer m, the output can be expressed as:

$$\begin{cases} \mathbf{y}_m = f(\mathbf{u}_m) \\ \mathbf{u}_m = \sum_{a \in L_{m-1}, b \in L_m} w_{ab} y_{m-1}^a + b_m \end{cases} \tag{11}$$

According to the chain rule defined by Equation 12

$$\frac{\partial L(w_{ij})}{\partial w_{ij}} = \frac{\partial L(w_{ij})}{\partial \mathbf{y}_m} \frac{\partial \mathbf{y}_m}{\partial \mathbf{u}_m} \frac{\partial \mathbf{u}_m}{\partial w_{ij}} \tag{12}$$

we further deduce the partial derivatives of $L(w_{11}, w_{12}, ..., w_{ij}, ...w_{mn})$ as in Equation 13, 14 and 15:

$$\begin{aligned} \frac{\partial L(wij)}{\partial \mathbf{y}_m} &= \frac{\partial \left(\frac{1}{2} (\mathbf{o}_m - \mathbf{y}_m)^2 \right)}{\partial \mathbf{y}_m} \\ &= \frac{1}{2} \times 2 (\mathbf{o}_m - \mathbf{y}_m) \frac{\partial (\mathbf{o}_m - \mathbf{y}_m)}{\partial \mathbf{y}_m} \\ &= -(\mathbf{o}_m - \mathbf{y}_m) \end{aligned} \tag{13}$$

$$\frac{\partial \mathbf{y}_{\mathrm{m}}}{\partial \mathbf{u}_m} = \frac{\partial f(\mathbf{u}_m)}{\partial \mathbf{u}_m} = f'(\mathbf{u}_m) \tag{14}$$

$$\begin{aligned} \frac{\partial u_m}{\partial w_{ij}} &= \frac{\partial (\sum_{a \in L_{m-1}, b \in L_m} w_{ab} y_{m-1}^a + b_m)}{\partial w_{ij}} \\ &= \frac{\partial (w_{11} y_{\mathrm{m-1}}^1 + w_{21} y_{\mathrm{m-1}}^2 + ... + w_{21} y_{\mathrm{m-1}}^2 + w_{\mathrm{ij}} y_{\mathrm{m-1}}^{\mathrm{i}} + ...)}{\partial w_{ij}} \\ &= y_{m-1}^i \end{aligned} \tag{15}$$

$\frac{\partial \mathbf{y}_{\mathrm{m}}}{\partial \mathbf{u}_m}$ is determined by the property of activation function. For $\frac{\partial u_m}{\partial w_{ij}}$, y_{m-1}^i is actually the i-th input of layer m-1.

Therefore, we get $\frac{\partial L(w_{ij})}{\partial w_{ij}} = -(\mathbf{o}_m - \mathbf{y}_m)\ f'(\mathbf{u}_m) y_{m-1}^i$,which is used to update the weight w_{ij}.

S4: Derivation of coefficients updating in MLP with L2 regularization

In order to deal with overfitting, we introduce L2 regularization in the manuscript where the loss function in Equation 10 is changed into Equation 16.

$$L(w_{11}, w_{12},..., w_{ij},...w_{mn}) = \frac{1}{2}(\mathbf{o}_i - \mathbf{y}_i(w_{11}, w_{12},..., w_{ij},...w_{mn}))^2 + \lambda \sum_{i \in L_{m-1}, j \in L_m} w_{ij}^2 \qquad (16)$$

The regularization parameter λ is used to limit the variation range of coefficients w_{ij}, so that it helps to prevent the complexity of deep learning model. After adding the regularization parameter, the derivation process of the parameter estimation and update is described as follows. According to Equation 13, 14 and 15, we further deduce the partial derivatives of Equation 16 as follows:

$$\begin{aligned} \frac{\partial L(w_{ij})}{\partial w_{ij}} &= \frac{\partial L(w_{ij})}{\partial \mathbf{y}_m} \frac{\partial \mathbf{y}_m}{\partial \mathbf{u}_m} \frac{\partial \mathbf{u}_m}{\partial w_{ij}} + \frac{\partial(\lambda \sum_{i \in L_{m-1}, j \in L_m} w_{ij}^2)}{\partial w_{ij}} \\ &= \text{-}(\mathbf{o}_m - \mathbf{y}_m) f'(\mathbf{u}_m) y_{m-1}^i + \frac{\partial(\lambda \sum_{i \in L_{m-1}, j \in L_m} w_{ij}^2)}{\partial w_{ij}} \\ &= \text{-}(\mathbf{o}_m - \mathbf{y}_m) f'(\mathbf{u}_m) y_{m-1}^i + 2\lambda w_{ij} \end{aligned} \qquad (17)$$

Hence, the update formula of parameter w_{ij} is changed as:

$$w_{ij} \leftarrow w_{ij} + \Delta w_{ij} = w_{ij} - \eta \frac{\partial L(w_{ij})}{\partial w_{ij}} = w_{ij} - \eta(\text{-}(\mathbf{o}_m - \mathbf{y}_m) f'(\mathbf{u}_m) y_{m-1}^i + 2\lambda w_{ij}) \qquad (18)$$
